# HPV self-sampling versus healthcare provider collection on the effect of cervical cancer screening uptake and costs in LMIC: a systematic review and meta-analysis

**DOI:** 10.1186/s13643-023-02252-y

**Published:** 2023-06-22

**Authors:** Selamawit F. Mekuria, Sydney Timmermans, Christer Borgfeldt, Mats Jerkeman, Pia Johansson, Ditte Søndergaard Linde

**Affiliations:** 1 Division of Oncology, Lund University, 22185 Lund, Sweden; 2 Department of Biomedical Sciences, University of Guelph, Guelph, Canada; 3 Department of Family Medicine, McGill University, Montreal, Canada; 4 Department of Obstetrics and Gynaecology, Lund University, Lund, Sweden; 5 School of Health and Welfare, Halmstad University, Halmstad, Sweden; 6 Department of Clinical Research, University of Southern Denmark, Odense, Denmark; 7 Department of Obstetrics and Gynaecology, Odense University Hospital, Odense, Denmark

## Abstract

**Background:**

Cervical cancer is a major global health issue, with 89% of cases occurring in low- and middle-income countries (LMICs). Human papillomavirus (HPV) self-sampling tests have been suggested as an innovative way to improve cervical cancer screening uptake and reduce the burden of disease. The objective of this review was to examine the effect of HPV self-sampling on screening uptake compared to any healthcare provider sampling in LMICs. The secondary objective was to estimate the associated costs of the various screening methods.

**Method:**

Studies were retrieved from PubMed, Embase, CINAHL, CENTRAL (by Cochrane), Web of Science, and ClinicalTrials.gov up until April 14, 2022, and a total of six trials were included in the review.

Meta-analyses were performed mainly using the inverse variance method, by pooling effect estimates of the proportion of women who accepted the screening method offered. Subgroup analyses were done comparing low- and middle-income countries, as well as low- and high-risk bias studies. Heterogeneity of the data was assessed using *I*^2^. Cost data was collected for analysis from articles and correspondence with authors.

**Results:**

We found a small but significant difference in screening uptake in our primary analysis: *RR* 1.11 (95% *CI*: 1.10–1.11; *I*^2^ = 97%; 6 trials; 29,018 participants). Our sensitivity analysis, which excluded one trial that measured screening uptake differently than the other trials, resulted in a clearer effect in screening uptake: *RR*: 1.82 (95% *CI*: 1.67–1.99; *I*^2^ = 42%; 5 trials; 9590 participants). Two trials reported costs; thus, it was not possible to make a direct comparison of costs. One found self-sampling more cost-effective than the provider-required visual inspection with acetic acid method, despite the test and running costs being higher for HPV self-sampling.

**Conclusion:**

Our review indicates that self-sampling improves screening uptake, particularly in low-income countries; however, to this date, there remain few trials and associated cost data. We recommend further studies with proper cost data be conducted to guide the incorporation of HPV self-sampling into national cervical cancer screening guidelines in low- and middle-income countries.

**Systematic review registration:**

PROSPERO CRD42020218504.

**Supplementary Information:**

The online version contains supplementary material available at 10.1186/s13643-023-02252-y.

## Introduction

### Background

Infection with a high-risk type of human papillomavirus (HPV) is the main cause of cervical cancer cases [1]. Cervical cancer is a disease that disproportionately affects women living in low- and middle-income countries (LMICs) [1, 2], given 89% of all cervical cancer cases in the world occur in these areas [3]. Furthermore, according to the World Health Organization (WHO), the total incidence and mortality of cervical cancer worldwide are estimated to increase in the upcoming years. LMICs will be the most severely affected, with cases predicted to double in certain areas of Africa and Asia [4]. Despite this, the resources actively invested in cervical cancer treatment worldwide are inversely related to the prevalence of the disease [5] — 94% of the global spending on cervical cancer treatment occurs in high-income countries (HICs) [3]. Hence, cervical cancer prevalence, diagnosis, and treatment are globally inequitable. Moreover, the prevalence and mortality of cervical cancer are one of the two major contributors to the increase in disability-adjusted life years (DALYs) [5] in LMICs.

Low cervical cancer screening is one of the main reasons for these health disparities [3]. Additionally, lack of knowledge and awareness about the disease, as well as psychological, structural, sociocultural, and religious factors, contribute to the low screening uptake [6]. In HICs, the successful reduction of cervical cancer incidence can be attributed to the high uptake of organized screening, as well as successful HPV vaccination programs [4]. Previously, screening in high-resource areas was mainly accomplished with cervical cytology examinations, conducted by healthcare professionals. However, in recent years, cytology has been replaced with HPV-based algorithms, such as primary HPV screening with reflex cytology. Research advancements have however found HPV analyses to be more sensitive in detecting high-grade intraepithelial lesions (HSIL) and cancer [7]. In low-resource settings, the WHO has long advocated the use of visual inspection with acetic acid (VIA) screen-and-treat programs [8]. However, the organization has recently shifted to primarily recommend HPV testing where possible, due to its higher sensitivity and the provider-dependency aspect of VIA [9]. In contrast to cytology samples, HPV samples can be collected by either a healthcare provider, or by the patient themselves, a so-called self-sample. Self-sampling for HPV has the advantage of being independent of location and direct involvement of health personnel, which is an important aspect in low-resource settings, and therefore may increase screening uptake [10]. The sample can be analyzed in a point-of-care (POC) setting or be collected and transported to a central laboratory for analysis. A positive HPV sample means that a woman has at least one of the 14 high-risk HPV genotypes as defined by the International Agency for Research on Cancer (IARC), i.e., 16, 18, 31, 33, 35, 39, 45, 51, 52, 56, 58, 59, 66, and 68 [11]. However, a positive sample needs to be followed by a triage — including a gynecological examination — to determine which patients may have precancerous lesions and thus require treatment. Hence, the cost of testing will vary depending on the intervention chosen and the laboratory techniques used.

It has been estimated that the use of both HPV-based and VIA-based screening as well as HPV vaccination in LMICs may decrease the number of cervical cancer cases by 5.2 million, avert 3.7 million deaths, and preserve 22.0 million DALYs [12]. Moreover, it has been suggested that HPV self-sampling is highly cost-effective in areas where screening coverage is relatively low (i.e., 15–20%), and that it may reduce cervical cancer incidence by approximately 20% [13]. There are several initiatives to introduce HPV self-sampling in LMICs. However, most of these have not been implemented in clinical routine. Hence, it is unclear whether self-sampling improves screening uptake in such settings compared to the use of provider-collected sampling. The acceptability of self-sampling was analyzed in a systematic review by Nelson et al., yet only three of the 37 studies they included were from low-resource settings [14]. One of their methods was the use of a proxy answer, where if a woman answered she would use the self-test again, it was interpreted that she was accepting of self-sampling. Nelsons et al.’s results concluded a high acceptability among the participants, but this excluded the three LMIC studies which had not reported on acceptability that could be used in their meta-analysis [14].

Moreover, a systematic review and meta-analysis from 2019 on the effect of HPV sampling versus provider-collected samples on screening uptake included studies from around the globe, with three out of 34 studies taking place in LMICs [15]. They found that HPV self-sampling improved screening uptake overall but more so in HICs (*RR*: 2.24; 95% *CI* 1.86–2.71; *I*^2^: 99.38) than in LMICs (*RR*: 1.54, 95% *CI* 1.01–2.34, *I*^2^: 98.43). More trials have been conducted in LMICs since 2019; hence, an updated review that solely focuses on LMICs may clarify the effect of HPV self-sampling in such settings. In 2021, a systematic review comparing self-sampling with clinician collected samples was conducted with RCTs from sub-Saharan African countries [16]. The aim was to examine implementation data according to a standard framework. Despite uptake being one of the study’s objectives and the title’ inclusion of the word meta-analysis, the important discussion that followed instead revolved around factors effecting implementation of a self-sampling-based screening system.

Furthermore, the testing costs of HPV self-samples may vary across countries, and cost represents a key component in the implementation of the technique in resource-limited settings. Therefore, cost remains another important factor to elucidate for improving future strategies to prevent cervical cancer in LMICs. There is thus a need to create a review that focuses on screening uptake and costs in LMICs, in order to find the gaps that need to be addressed and knowledge requiring implementation.

This review aims to synthesize the evidence of the effect of HPV self-sampling versus healthcare-provider screening methods for cervical cancer screening uptake in lower- and middle-income countries. Furthermore, our review seeks to summarize available cost data to describe the difference in resource consumption between the two types of screening services.

## Material and methods

### Protocol registration

The protocol for this systematic review was developed based on PRISMA-P guidelines and was registered on December 12, 2020, in PROSPERO (CRD42020218504) prior to study conduct.

### Eligibility criteria

Studies were selected based on the following criteria: participants, intervention, comparator, outcomes, study design, and setting (PICO-S).

Participants in the trials included women and transgender men with a cervix who were eligible for cervical cancer screening according to respective national cancer screening guidelines. No age limit was applied. Trials permitting individuals who had a prior history of cervical cancer were excluded. The intervention criteria included studies that used vaginal self-swabs for HPV testing (any form of vaginal self-collection device) as the index test. Trials that solely used provider collected sampling methods were excluded.

For the comparator criteria, we included studies that had a healthcare provider-collected screening method as the control group, e.g., cytology, VIA, or provider-collected HPV samples. There was no limitation to the precise location where the specimen collection occurred for the intervention or the control group.

The primary outcome was cervical cancer screening uptake, defined as the proportion of women attending primary cervical cancer screening. The secondary outcome was the difference in start-up and running costs (monetary and time) between the screening methods. We defined start-up costs as training of healthcare providers and analyzing instruments, whereas running costs were defined as healthcare providers’ salaries, transportation costs (by distance) for the screening clients, material for specimen collection (whether provider- or self-collected), and transportation costs for the specimen collected. If studies did not list costs but solely calculated screening uptake, they were included in the review.

The study design and setting criteria included randomized controlled trials — both single and cluster randomized — set in LMICs as defined by the World Bank Group [17].

### Search strategy and identification of studies

Electronic searches were completed by a university librarian (MB) according to a search strategy developed by M. B., S. T., D. S. L., and S. M. (supplemental file 1). The following databases were searched: PubMed, Embase, CINAHL, Cochrane’s CENTRAL, Web of science, Scopus, and ClinicalTrials.gov, up to April 14, 2022. No language or lower publication date restrictions were applied. If any studies required translation, the Lund University uses an external service that would have been consulted; however, this was not required. Furthermore, a manual search for relevant conference abstracts was conducted by ST on the following conferences: IPVS (International Papilloma Virus Society) 2018, IPVS 2020, Federation International of Gynecology and Obstetrics (FIGO) 2015, and FIGO 2018. If the conference abstracts met the inclusion criteria, they were eligible to be included in the review.

### Data collection

Search results from the different databases and other sources were combined in an EndNote library (Version X9.3.1; Thomson Reuters, New York, NY, USA), and any duplicate records were removed. The remaining literature was uploaded to Covidence (www.covidence.org), where a second duplicate check was made. Two authors independently verified inclusion and exclusion of eligible studies at the title and abstract level and discussed any discordance (S. M., S. T.). Two authors then independently repeated the same strategy for the full-text screening (S. M., C. B.). The following data was extracted from the included studies through an Excel template: title, participants, randomization method, intervention, control, outcomes, setting, and intra-cluster coefficients. One author (S. M.) contacted trial authors for missing or unpublished data.

### Assessment of risk of bias

Three authors (S. M., S. T., M. J.) assessed all studies according to the Cochrane Risk-of-Bias tool 2.0 (for single and cluster randomized trials) using the criteria outlined in the Cochrane Handbook of Systematic Reviews of Interventions [18]. The following domains were assessed: (1) selection bias, (2) performance bias, (3) detection bias, (4) attrition bias, and (5) reporting bias. The studies were concluded to have either a low, unclear, or high risk of bias. When there were disagreements, the authors discussed their reasoning, and a final vote decided the bias level.

### Data synthesis

The meta-analyses were carried out using Review Manager 5 software [19] comparing HPV self-sampling in the intervention and health provider screening in the control. Pooled RRs were calculated, and 95% confidence intervals (CIs) were estimated using the inverse variance method instead of a random-effects model due to the inclusion of cluster-randomized trials in the primary analysis. A sensitivity analysis was performed using the same statistical method, except when we excluded RCTs and then instead used the random-effects model (Mantel–Haenszel method for dichotomous data). To avoid overestimating the effect of the intervention, clustering was taken into account for the meta-analyses, and if studies did not report an intra-cluster coefficient, 0.098 was used to calculate the effective sample size, as according to the Cochrane Handbook [18]. Statistical heterogeneity was assessed by using *I*^2^ [18]. Subgroup analyses were conducted comparing low-income countries to middle-income countries. Furthermore, we compared low-risk-of-bias trials with high-risk-of-bias trials. Low-risk bias trials were defined as trials with a low risk of selection bias, detection bias, and reporting bias. All other trials were viewed as having a high-risk bias. Cost data was analyzed descriptively as it was not possible to conduct a direct comparison of costs due to differing data types.

### Patient and public involvement

Patients and the public were not involved in the planning and conduction of this study. However, since the majority of this study’s authors have other research work in low-income settings, the plan is to discuss the results with stakeholders in these countries.

## Results

### Summary of included studies

**Fig. 1:**
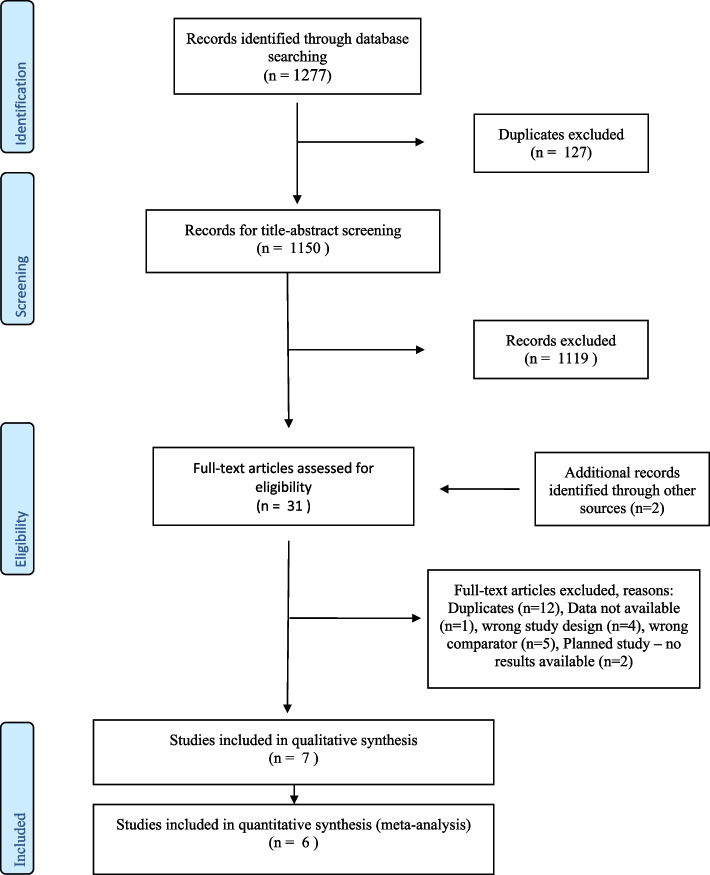
PRISMA flow chart

A total of 1163 records were found during the electronic database search, and two records were found through other sources. Among these, 127 were duplicates, and 1036 records were title-abstract screened, while 30 articles were read in full text. A total of six trials published in seven different papers were included in the review [20–26] (Fig. 1). One trial published a cost-effectiveness analysis in a separate paper, and this was used for the descriptive cost analysis.

### Characteristics of studies

The included studies were from countries in sub-Saharan Africa and North/South America, specifically Uganda, Nigeria, Ethiopia, Mexico, Brazil, and Argentina [20–22, 24–26] (Table 1). Overall, the countries’ screening coverage — as reported by the WHO — varied. Out of the six countries, only four had initiated national screening programs for cervical cancer to the extent that they could share screening coverage [27]. The trials were published between 2011 and 2019 and contained a total number of 24,633 women (with no noted transgender participants). The participants’ ages ranged between 25 and 65 years, and the studies included both rural and urban populations. Three trials were cluster trials [21, 24, 26]. The recruitment of participants in four of the studies was completed using community health workers (CHWs) through outreach programs [20, 22, 24, 26]. Each of the studies used a different HPV self-sampling device (see Table 1). The control group used either cytology, VIA, or an HPV vaginal test collected by a healthcare provider [20–22, 24–26]. Five studies listed uptake of screening or adherence to the procedure as their primary outcome [21, 22, 24–26]. In Lazcano et al., the primary outcome was the relative sensitivity and positive predictive values of HPV self-sampling in comparison with a provider-collected HPV sample, yet the study reported results on screening uptake for each arm and was therefore found eligible to be included in the review [20].

**Table 1:** Included studies’ characteristics and summary statistics

Study	Setting	Participants	Method of randomization	Intervention/control	Uptake HPV self-sampling	Uptake healthcare provider screening	Cost outcome
Arrossi 2015 [26]	Urban and rural areas in Argentina	6013 women from age 30 and above	Cluster	1. HPV self-sampling (cervical brush Qiagen) 2. Cytology and HPV healthcare provider collected sample	2552/3049 (83.7%)	599/2964 (20.2%)	None provided
Castle 2019 [24]	Town of Maringa in Brazil	483 women. Age 25–64	Cluster	1. HPV self-sampling (brush “just for me”) 2. Cytology 3. A choice between 1 & 2 HPV cobas analysis	161/161 (100%)	96/160 (60.0%)	None provided
Gizaw 2019 [21]	Butajira region, Ethiopia. Urban and rural	Women included in the Butajira Health and Demographic Surveillance platform between age 30 and 49	Cluster	1. HPV self-sampling (Evalyn brush) 2. VIA	1020/1213 (84.1%)	575/1143 (50.5%)	Running costs - Intervention and control arm US $267.50/month (community health worker salary) - Control arm and HPV-positive women: US $200.62/month (screening nurse salary) - Participant’s travel allowance US $6.69/person
Lazcano 2011 [20]	Rural Mexico	21,742 women. Age 25–64	Single	1. HPV self-sampling (Diegene conical swab) 2. Pap smear Hybrid capture analysis	9202/9371 (98.2)	9761/10,057 (97.1)	None provided
Modibbo 2017 [25]	Semi-urban districts of Abuja, Nigeria	400 women. Age 30–65	Single	1. HPV self-sampling 2. Health provider collected HPV sample Dry flocked swab GP5 + /6 + PCR-EIA system with LMNX genotyping	185/200 (92.5)	113/200 (56.5%)	None provided
Moses 2015 [22] Mezei 2018 [23]	Kisenyi district (urban), Kampala, Uganda	500 participants. Age 30–65	Single	1. HPV vaginal self-collection (Dacron swab) 2. VIA	248/250 (99.2%)	121/250 (48.4%)	Start-up costs - Training of health worker + community campaign US $6.58 - Lab equipment is included in lab cost Running costs - Healthcare workers’ time cost/woman screened. Intervention: US $5.50, control: US $8.25 - Time spent by women (transportation, waiting, procedure): intervention: 25 min, control: 180 min + 30 min with cryotherapy - Time cost for women: HPV self-collection US $0.21. VIA screen US $1.53 - Material pr. test intervention: US $6.70. Control: US $0.79 Laboratory cost: per HPV test: US $0.69 - Midwife costs (VIA + /cryotherapy) US $14.26 (VIA +). US $5.67 (VIA −)

### Secondary outcome

One trial had published a cost-effectiveness analysis (CEA) based on their trial in a separate paper, which was included in the review for the cost analysis [23]. Furthermore, through email correspondence, some cost data was provided by Gizaw et al. [21]. The remaining trials provided no cost data. The cost estimates varied to an extent that a direct comparison between the studies was not possible.

The summarized cost data was converted to a year 2020 USD using the Campbell and Cochrane Economics Methods Group (CCEGM) currency converter [28]. In Mezei, the intervention demonstrated a cost of US $13.10 per woman, which was lower than the control strategy that used VIA with a cost of US $16.24 [23]. For the women that were HPV positive or in the control arm, an additional cost of US $14.26 (VIA +)/US $5.67(VIA −) or US $8.59 (VIA +) respectively was needed. The total cost of each strategy included CHW’s cost at inclusion, material, and the cost of midwife to carry out VIA. Additionally, the women spent less time (e.g., for transportation) in the self-sampling arm than in the VIA arm. Based on the cost analysis, the authors concluded that community-based HPV self-sampling followed by treatment of all HPV-positive women had the potential to be both a preventative and cost-effective screening strategy [23].

In Gizaw et al., the cost per month of a CHW, who informs the woman of the screening method, was US $267.50, whereas those randomized to VIA had an additional cost of US $200.62 per month [21]. This VIA cost also applies to those HPV positive, but since they are a minority, the total cost for the HPV self-sampling strategy seems lower from the sparse data received from the author.

### Risk-of-bias assessment

**Fig. 2:**
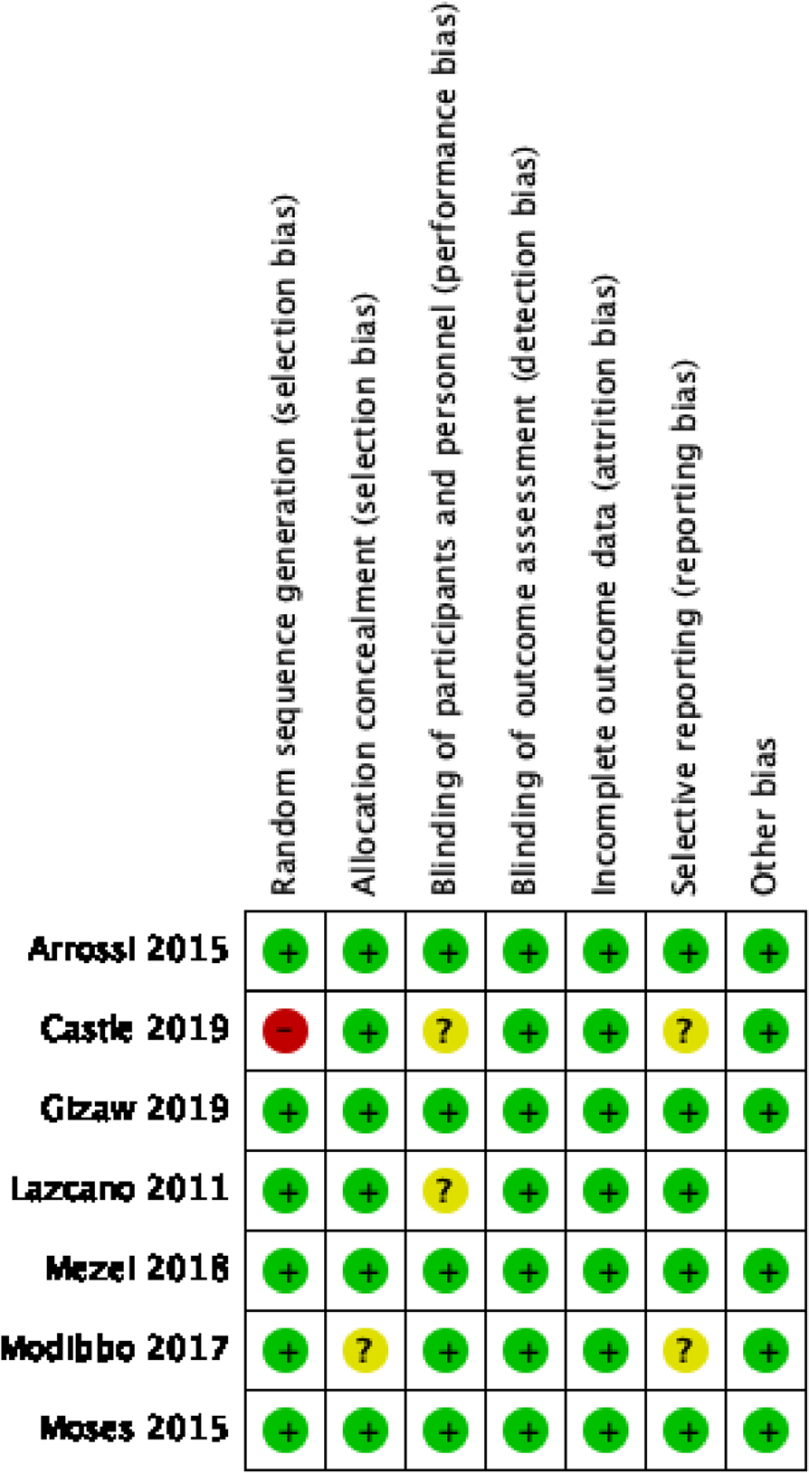
Risk-of-bias assessment for all included studies

All studies included in the meta-analysis were assessed for risk of bias (Fig. 2). Most of the studies used some type of random sequence generation for the allocation of participants. Two studies, Castle and Modibbo, received high risk of bias and “some concern” respectively for this domain. Regarding cluster randomized trials, we discussed baseline imbalances, which none of the studies demonstrated. Blinding to the intervention was not feasible in any of the studies, as the participants needed to be informed of their screening activity. Instead, different methods to minimize contamination between the study groups were used. Those methods included the use of a buffer zone between two villages, a pre-specified lists of participants allocated beforehand to each intervention group, the use of sealed envelopes with intervention details only to be opened after participant recruitment, and blinding community health workers to the presence of another intervention. We deemed a study as having a low risk of performance bias if the study employed any of the above tactics. All studies were assessed as having a low risk of attrition bias because they all presented the prespecified outcome data, usually using flow charts. A study was deemed to have low-risk bias for selective reporting if there was a prespecified plan or published protocol to be found. Of the included studies, four were deemed to have a low risk of reporting bias.

Overall, three studies were denoted as having a low risk of bias, one study was noted as having a high risk of bias, and three studies were assessed as having some concerns.

### Meta-analysis

Six trials were included in the meta-analysis, and the data can be found in the appendix. Two out of three cluster trials did not take clustering into account in their analysis [21, 24]. Arrossi et al. did take clustering into account; however, they included women who accepted self-sampling but then later refused to give a sample [26], which we regarded as not adhering to the intervention. Hence, we ended up calculating the effective sample size for three cluster trials before conducting the meta-analysis (supplementary file 2).

**Fig. 3:**
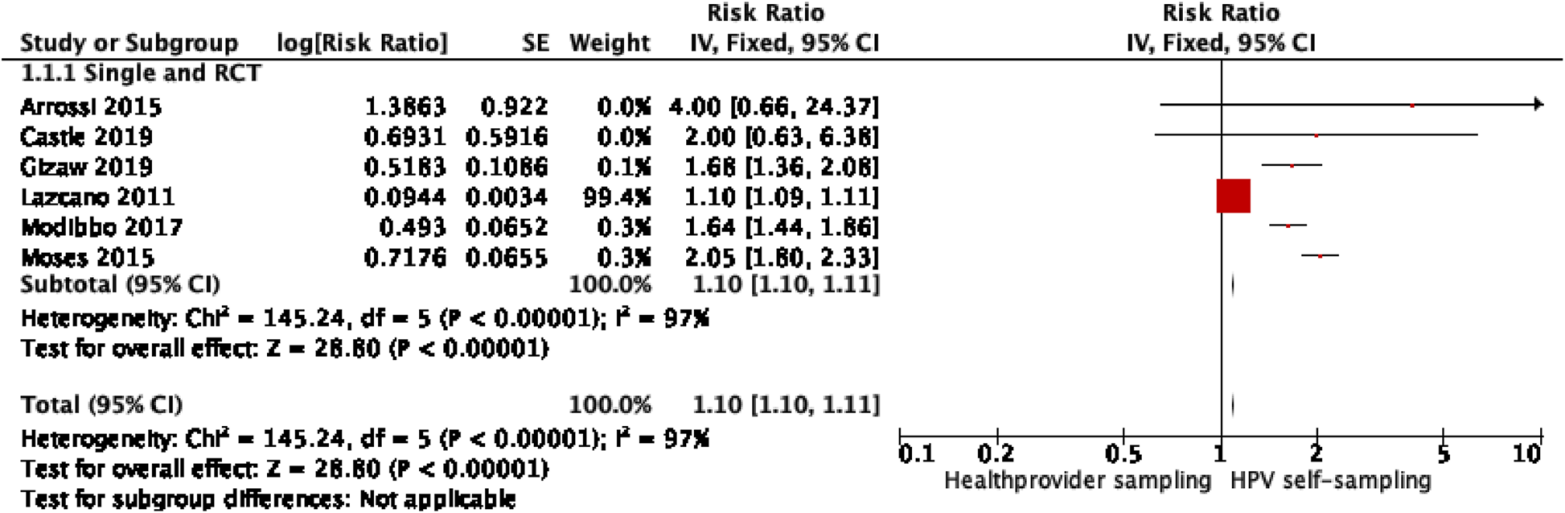
Effect of self-sampling versus healthcare provider screening (control) in LMIC

**Fig. 4:**
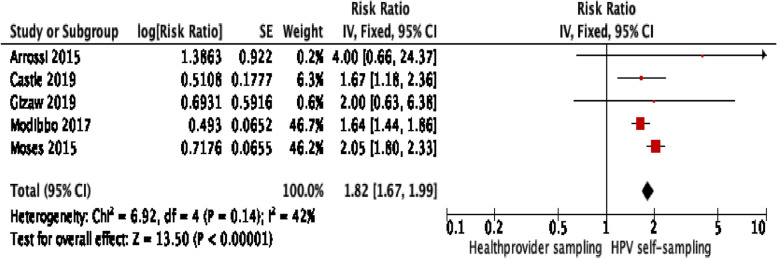
Sensitivity analysis — excluding Lazcano

We found a significant difference in uptake of cervical cancer screening between HPV self-sampling and the control group, *RR*: 1.11; 95% *CI*: [1.10–1.11], *I*^2^ = 97% (Fig. 3). However, heterogeneity was high, and the results were primarily driven by the large-scale trial by Lazcano et al., which primary outcome was to measure the sensitivity of HPV self-sampling [20]. As a result, the screening uptake was depicted according to their primary outcome of interest. For example, in this study, the women who were randomized to the HPV self-sampling arm (the intervention group) but were not found at home upon inclusion were excluded. However, women who were allocated to the provider-collected cytology test (the control group) but not found at home upon inclusion were still considered eligible for the trial and invited for a scheduled appointment to be enrolled into the study (see figure “trial profile” in [20]). Due to their different outcome, the Lazcano et al. trial may skew the overall results of the meta-analysis, and thus, we decided to exclude this trial from the post hoc sensitivity analysis. The sensitivity analysis found a higher effect of HPV self-sampling on screening uptake, and heterogeneity improved greatly, *RR*: 1.82; 95% *CI* [1.67–1.99], *I*^2^ = 42% (Fig. 4).

**Fig. 5:**
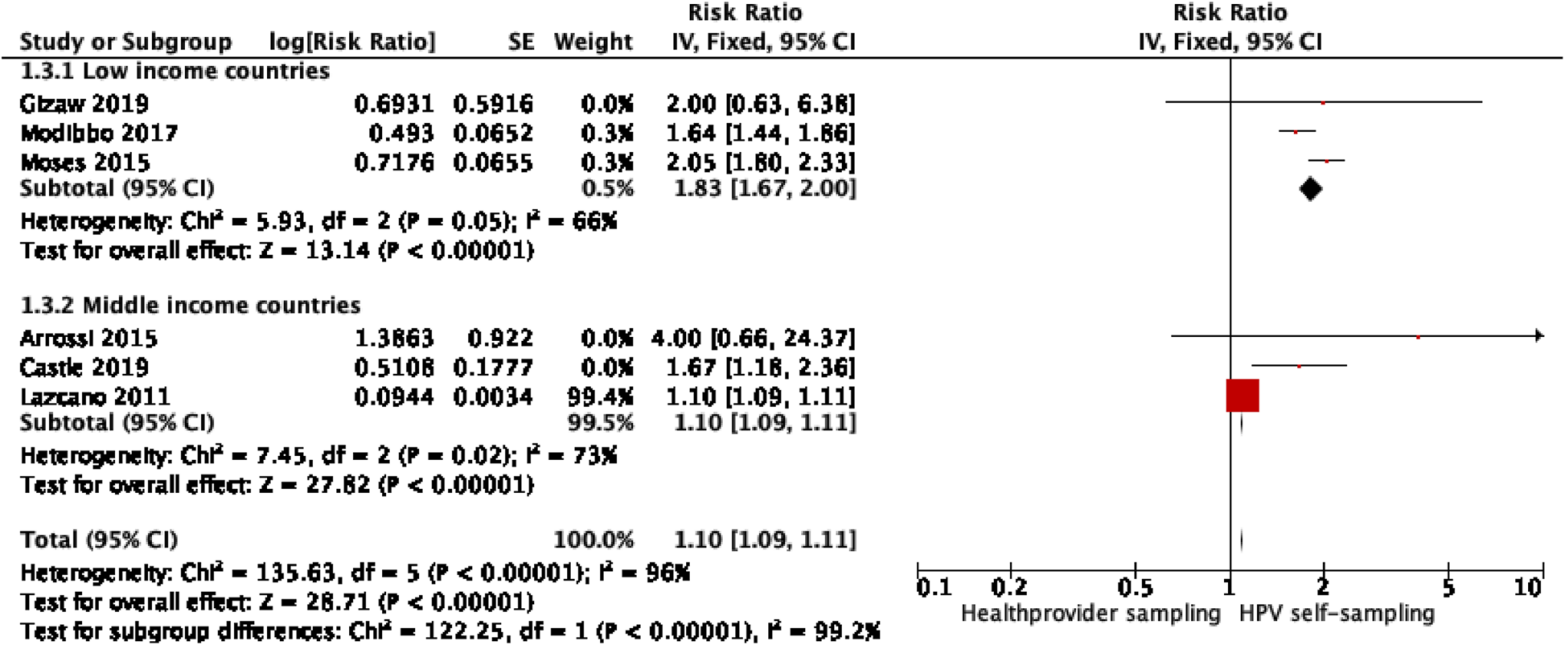
Subgroup analysis. Low-income versus middle-income countries (with Lazcano)

The comparison of low-income versus middle-income countries in the meta-analysis reflected the same results as comparing the three WHO regions [17] — Africa and North/South America. Three trials were carried out in low-income countries, i.e., Ethiopia, Uganda, and Nigeria [21, 22, 25], and three trials took place in middle-income countries, i.e., Mexico, Argentina, and Brazil [20, 24, 26]. One subgroup analysis depicted that HPV self-sampling improved cervical cancer screening uptake more so in low-income countries compared to middle-income countries, *RR*_low_: 1.83; 95% *CI* (1.67–2.00), *I*^2^ = 66% versus *RR*_middle_: 1.10; 95% *CI* (1.09–1.11), *I*^2^ = 73%, and that this difference was significant; *p* < 0.0001 (Fig. 5). The low-income country subgroup analysis presented a high but improved heterogeneity compared to the middle-income countries group.

**Fig. 6:**
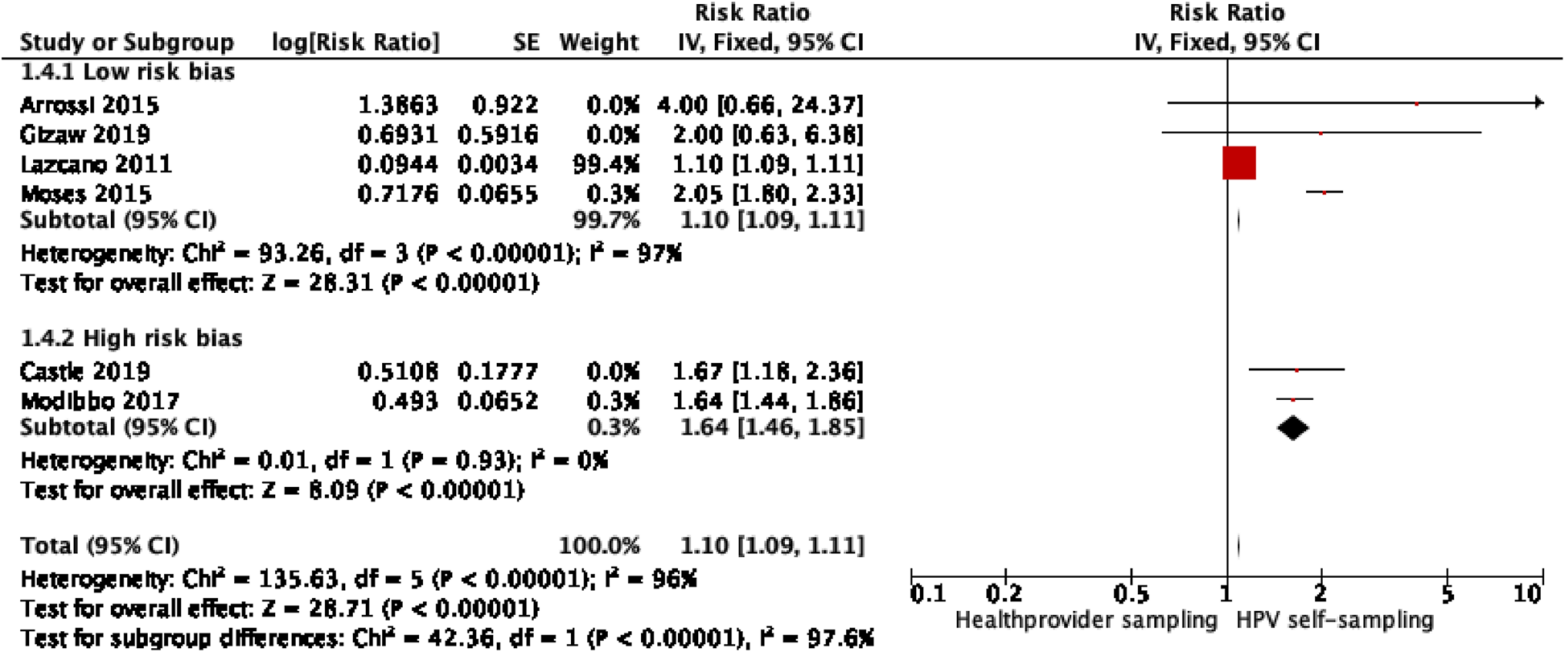
Subgroup analysis — high-risk versus low-risk-of-bias studies (with Lazcano)

Four trials were assessed as overall low-risk bias [20, 21, 26], and two trials were deemed as overall high-risk bias [22, 24]. Another subgroup analysis could demonstrate that high-risk bias trials had a significantly greater uptake and improved heterogeneity in the HPV-self-sampling arm 1.64; 95% *CI* (1.46–1.85), *I*^2^ = 0% in comparison with low-risk-of-bias studies 1.10; and 95% *CI* (1.09–1.11), *I*^2^ = 97% *p* < 0.00001 (Fig. 6).

## Discussion

This systematic review and meta-analysis of six trials set in LMICs found that HPV self-sampling increased screening uptake compared to different types of health-provider screening methods. Four out of the six trials were judged to have a low risk of bias, and overall heterogeneity was high. Furthermore, there was a lack of cost data, and it was therefore not possible to make a proper cost analysis across all trials. The cost data that was available reported that self-sampling saved women time and had lower costs associated with healthcare personnel, despite operating and test costs being higher than VIA.

Overall, there was a limited number of trials eligible for inclusion into this review, which limits the generalizability of our results. Upon further review, Lazcano et al. was additionally excluded from the sensitivity analysis, because the trial’s main objective was not to measure uptake [20]. The inclusion of this trial in our sensitivity analysis would have greatly affected our main analysis, due to level of heterogeneity the trial introduced to our data. However, we still decided to include the trial in our review and in our main analysis to maintain transparency, as it fulfilled inclusion criteria outlined in our protocol.

The subgroup analysis demonstrated that the effect of self-sampling was significantly greater in the low-income countries (*RR*_low_: 1.83; 95% *CI*: 1.67–2.00, *I*^2^ = 66% versus *RR*_middle_: 1.10; 95% *CI* 1.09–1.11, *I*^2^ = 73%; *p* < 0.0001). The heterogeneity remained high but decreased in both subgroups, which suggests a more uniform effect in these regions. Overall, we found high heterogeneity across the studies, which may not only be due to the different study settings but also the low number of studies included in the meta-analysis, recruitment variations, differing HPV self-sampling devices, and various healthcare-provider collection methods. More trials taking place in LMICs are needed to better estimate the true effect of self-sampling on screening uptake.

The low-income countries included were all from sub-Saharan Africa, a region which commonly uses opportunistic screening methods alongside VIA. However, in 2021, the WHO updated its screening guidelines to now recommend HPV testing as the primary screening method in all settings [1]. Hence, a shift away from VIA and towards HPV tests for screening will most likely be seen in sub-Saharan Africa in the years to come. Cervical screening uptake has historically been particularly low in sub-Saharan Africa [29], and HPV self-sampling has the potential to address a number of the underlying factors that cause low uptake. One Ethiopian study found that a lack of resource accessibility has been found to cause low uptake [30], while a study from Tanzania found that fear of gynecological examination, transportation costs to the clinic, and wait times at the clinic were factors for women not to attending screening [31]. Furthermore, factors affecting uptake of cervical cancer screening were summarized by Devarapalli et al. (2018), where structural barriers (i.e., time), psychological barriers (i.e., modesty), lack of knowledge of the disease and its treatments, and sociocultural barriers (i.e., lack of family support) were described as reasons for the low screening uptake in LMICs [6]. Hence, implementation of HPV self-sampling may improve screening uptake but requires education and awareness about the disease for both women and their families to be successful.

The cost data extracted from our included studies is scarce. Only Mezei et al. described the cost difference between the HPV self-sampling arm and the VIA arm, with the latter being more costly mainly because of the greater cost of human resources (Table 1), despite material and laboratory costs being higher for the self-sampling arm compared to VIA. Additionally, the time required by the participant for VIA is nine times longer than that of the self-sampling arm. Only those found to be HPV positive in the self-sampling arm would have to spend a similar amount of time as VIA participants [23]. Therefore, it appears as though transportation time as well as wait times decrease for women in the self-sampling arm, according to the Mezei et al. (see Table 1).

VIA may thus no longer be a viable screening method to compare with HPV testing when discussing future screening strategies for low-income settings.

The lack of cost data from the studies captured in our search strategy herein caused us to seek cost data from other studies not previously included. A health economics evaluation by Flores et al. in 2011 [32] out of Mexico was found after the search process because cost-effectiveness was not a part of our protocol’s search strategy, as we planned to extract cost data as a secondary outcome from the RCTs we found. In Flores et al., all associated costs with regard to different screening methods were presented, and their analysis showed that the average total cost of HPV self-sampling was lower in comparison with both cytology and healthcare provider-collected HPV sampling [32]. This finding is similar to the Mezei et al. trial reporting [23]. Moreover, a recent meta-analysis by Malone et al. (2020) concluded that self-sampling is cost-effective if the screening uptake is high (i.e., over 20%), yet there is a lack of cost-effectiveness analyses from LMICs and vulnerable populations in general [33]. Hence, a discussion on how to interpret the results from our review needs to be based on further studies about resource consumption and cost-effectiveness of HPV self-sampling in LMICs.

The major limitations of this systematic review include the limited number of studies that were available, including the lack of RCTs from other parts of the world, such as Middle East and Asia. Another limitation is the scarcity of cost data that we were unable to retrieve from the authors despite efforts, which renders our cost comparison weak. However, the strength of our study is the discussion that arises about why HPV self-sampling may differ between low-income and middle-income countries. Additionally, our decision to include cost data as a secondary outcome also represents a strength, as it highlights the lack of cost information with regard to HPV self-sampling in LMICs. The limited availability of cost data served to reinforce the statement from Malone et al. that more evidence regarding the cost-effectiveness of self-sampling based in LMICs is needed, given the crucial fact that screening uptake and cost-effectiveness go hand in hand [33]. In summary, the combination of these factors may prove valuable for local stakeholders in LMICs and help in the implementation strategy of primary-based HPV self-sampling for cervical cancer screening.

## Conclusion

HPV self-sampling significantly improves cervical cancer screening uptake in low-income countries in comparison with middle-income countries and has potential to assist in reducing the global burden of cervical cancer. However, more research is needed to better understand the effect of self-sampling on screening uptake across low- and middle-income settings, specifically with studies done to evaluate uptake of this screening form as the primary outcome. There is a scarcity of cost data regarding this screening method, yet the limited data that was found suggested that HPV self-sampling may be a cost-effective screening strategy in such settings. However, future cost evaluating studies are required to better inform national cervical cancer screening guidelines and allow for HPV self-sampling implementation in future cancer surveillance plans.

## Supplementary Information

**Additional file 1.** Appendix. Final search strategy for PubMed.

**Additional file 2.** Risk-of-bias HPV summary.

**Additional file 3.** Raw data HPV.

**Additional file 4.** Sensitivity analysis — excluding cluster RCT.

## Data Availability

All data generated or analyzed during this study are included in this published article [and its supplementary information files].

## Abbreviations

LMIC: Low- and middle-income countries
HPV: Human papillomavirus
WHO: World Health Organization
HIC: High-income countries
DALY: Disability-adjusted life years
HSIL: High-grade intraepithelial lesion
VIA: Visual inspection with acetic acid
POC: Point of care (test)
IARC: International Agency for Research on Cancer
CCEGM: Campbell and Cochrane Economics Methods Group
RCT: Randomized controlled trials
