# Supplementary material for: HPV self-sampling versus healthcare provider collection on the effect of cervical cancer screening uptake and costs in LMIC: a systematic review and meta-analysis
Source: Syst Rev. 2023 Jun 22;12:103. doi: 10.1186/s13643-023-02252-y (PMC10286394; doi:10.1186/s13643-023-02252-y)
Supplement: Supplementary file 1 — Additional file 1. Appendix. Final search strategy for PubMed. [file 13643_2023_2252_MOESM1_ESM.pdf]

Appendix

Final search strategy for PubMed
No language or publication date restrictions were applied. A modified Cochrane filter for randomized studies was applied, together with a filter for LMICs developed by Ditte Søndergaard Linde et al for a previous systematic review.

((("human papillomavirus"[MeSH Terms]) OR "human papilloma virus" OR "human papillomavirus" OR hpv OR papillomaviridae) AND (self sampl* OR self collect* OR self swab* OR self test* OR home based test OR home test OR self taken OR CareHPV OR participant collected sampling OR self-admin* OR self-sampl* OR self-collect* OR self-swab* OR self-test* OR home-based test OR home-test OR self-taken OR participant-collected sampling)))
AND
(("Developing Countries"[MeSH] OR Deprived Countries OR Deprived Population OR Deprived Populations OR Developing Countries OR Developing Country OR Developing Economies OR Developing Economy OR Developing Nation OR Developing Nations OR Developing Population OR Developing Populations OR Developing World OR LAMI Countries OR LAMI Country OR Less Developed Countries OR Less Developed Country OR Less Developed Economies OR Less Developed Nation OR Less Developed Nations OR Less Developed World OR Lesser Developed Countries OR Lesser Developed Nations OR LMIC OR LMICS OR Low GDP OR Low GNP OR Low Gross Domestic OR Low Gross National OR Low Income Countries OR Low Income Country OR Low Income Economies OR Low Income Economy OR Low Income Nations OR Low Income Population OR Low Income Populations OR Lower GDP OR lower gross domestic OR Lower Income Countries OR Lower Income Country OR Lower Income Nations OR Lower Income Population OR Lower Income Populations OR Middle Income Countries OR Middle Income Country OR Middle Income Economies OR Middle Income Nation OR Middle Income Nations OR Middle Income Population OR Middle Income Populations OR Poor Countries OR Poor Country OR Poor Economies OR Poor Economy OR Poor Nation OR Poor Nations OR Poor Population OR Poor Populations OR poor world OR Poorer Countries OR Poorer Economies OR Poorer Economy OR Poorer Nations OR Poorer Population OR Poorer Populations OR Third World OR Transitional Countries OR Transitional Country OR Transitional Economies OR Transitional Economy OR Under Developed Countries OR Under Developed Country OR under developed nations OR Under Developed World OR Under Served Population OR Under Served Populations OR Underdeveloped Countries OR Underdeveloped Country OR underdeveloped economies OR underdeveloped nations OR underdeveloped population OR Underdeveloped World OR Underserved Countries OR Underserved Nations OR Underserved Population OR Underserved Populations OR Afghanistan OR Albania OR Algeria OR "American Samoa" OR Angola OR Argentina OR "Argentine Republic" OR Armenia OR Azerbaijan OR Bangladesh OR Belarus OR Byelarus OR Belorussia OR Belize OR Benin OR Bhutan OR Bolivia OR Bosnia OR Botswana OR Brazil OR Bulgaria OR Burma OR "Burkina Faso" OR Burundi OR "Cabo Verde" OR "Cape verde" OR Cambodia OR Cameroon OR "Central African Republic" OR Chad OR China OR Colombia OR Comoros OR Comores OR Comoro OR Congo OR "Costa Rica" OR "Côte d'Ivoire" OR Cuba OR Djibouti OR Dominica OR "Dominican Republic" OR Ecuador OR Egypt OR "El Salvador" OR Eritrea OR Ethiopia OR Fiji OR Gabon OR Gambia OR Gaza OR "Georgia Republic" OR Georgian OR Ghana OR Grenada OR Grenadines OR Guatemala OR Guinea OR "Guinea Bissau" OR Guyana OR Haiti OR Herzegovina OR Hercegovina OR Honduras OR India OR Indonesia OR Iran OR Iraq OR Jamaica OR Jordan OR Kazakhstan OR Kenya OR Kiribati OR Korea OR Kosovo OR Kyrgyz OR Kirghizia OR Kirghiz OR Kirgizstan OR Kyrgyzstan OR "Lao PDR" OR Laos OR Lebanon OR Lesotho OR Liberia OR Libya OR Macedonia OR Madagascar OR Malawi OR Malay OR Malaya OR Malaysia OR Maldives OR Mali OR "Marshall Islands" OR Mauritania OR Mauritius OR Mexico OR Micronesia OR Moldova OR Mongolia OR Montenegro OR Morocco OR Mozambique OR Myanmar OR Namibia OR Nauru OR Nepal OR Nicaragua OR Niger OR Nigeria OR Pakistan OR Palau OR Panama OR "Papua New Guinea" OR Paraguay OR Peru OR Philippines OR Phillippines OR Philipines OR Phillipines OR Principe OR Romania OR Rwanda OR Ruanda OR Samoa OR "Sao Tome" OR Senegal OR Serbia OR "Sierra Leone" OR "Solomon Islands" OR Somalia OR "South Africa" OR "South Sudan" OR

"Sri Lanka" OR "St Lucia" OR "St Vincent" OR Sudan OR Surinam OR Suriname OR Swaziland OR Syria OR "Syrian Arab Republic" OR Tajikistan OR Tadzhikistan OR Tadjikistan OR Tadzhik OR Tanzania OR Thailand OR Timor OR Togo OR Tonga OR Tunisia OR Turkey OR Turkmen OR Turkmenistan OR Tuvalu OR Uganda OR Ukraine OR Uzbek OR Uzbekistan OR Vanuatu OR Venezuela OR Vietnam OR "West Bank" OR Yemen OR Zambia OR Zanzibar OR Zimbabwe))
AND
(((("randomized controlled trial"[Publication Type]) OR ("controlled clinical trial"[Publication Type])) OR (randomized[Title/Abstract] OR randomised[Title/Abstract] OR randomly[Title/Abstract] OR trial[Title/Abstract] OR groups[Title/Abstract] OR cluster*[Title/Abstract]))
