# Supplementary material for: HPV self-sampling versus healthcare provider collection on the effect of cervical cancer screening uptake and costs in LMIC: a systematic review and meta-analysis
Source: Syst Rev. 2023 Jun 22;12:103. doi: 10.1186/s13643-023-02252-y (PMC10286394; doi:10.1186/s13643-023-02252-y)
Supplement: Supplementary file 2 — Additional file 2. Risk-of-bias HPV summary. [file 13643_2023_2252_MOESM2_ESM.pdf]

Moddibo et al, 2017 Nigeria **Risk of bias:** some concern

Bias	Authors' judgement	Support for judgement
Domain 1<br>Randomization<br>(selection bias)	Some concern	No informtion how the allocation was given to each participant
Domain 2<br>Deviations from the intended intervention/Blinding<br>(Performance bias)	Low	Intention to An intention to treat analysis was used
Domain 3<br>Incomplete outcome data<br>(Attrition bias)	Low	All data for the outcome uptake was present
Domain 4<br>Measurement of outcome<br>(detection bias)	Low	Dichotomous data, proportions used
Domain 5<br>Selective reporting<br>(reporting bias)	Some concern	No information on a prescpecified plan was found, nor a published protocol

Arrossi et al, 2015 Argenti Cluster-RCT **Risk of Bias:** low

Bias	Authors' judgement	Support for judgement
Domain 1<br>Randomization<br>(selection bias)	Low	CHW were first stratified then randomized to each cluster<br>and then to intervention.
Identification of participants	Low	The CHW had no option to select one intervention over another
Domain 2<br>Blinding of participants and personnel/<br>(performance bias)	Low	They don't mention consent, hence we assume the participants<br>were not aware of the trial, bc the methods are normal<br>practice in this region
Domain 3<br>Incomplete outcome data<br>(attrition bias)	Low	No missing data
Domain 4<br>Blinding of outcome assessment<br>(detection bias)	Low	The outcome was not recorded and calculated<br>by the same person
Domain 5<br>Selective reporting<br>(reporting bias)	Low	Protocol published

Moses et al 2015, Uganda **Risk of bias:** low

Bias	Authors' judgement	Support for judgement
Domain 1<br>Randomization<br>(selection bias)	Low	Use of simple computer-generated simple randomization and envelopes
Domain 2<br>Blinding of participants and personnel/<br>(performance bias)	Low	Yes, they were aware. Intention to treat analysis was made
Domain 3<br>Incomplete outcome data<br>(attrition bias)	Low	No incomplete data
Domain 4<br>Blinding of outcome assessment<br>(detection bias)	Low	Outcome assessors were aware, but outcome was not effected.<br>Use of proportion
Domain 5<br>Selective reporting<br>(reporting bias)	Low	Protocol was published

Castle et al, 2019 Brazil **Risk of Bias:** high

Cluster RCT

Bias	Authors' judgement	Support for judgement
Domain 1<br>Randomization<br>(selection bias)	High	No description of randomisation process.<br>Only the word "randomized" was used.
Identification of participants	Low	They were identified before the assignment to intervention
Domain 2<br>Blinding of participants and personnel/<br>(performance bias)	Some concerns	No information on what analysis was mde<br>to estimate the effect of assignment
Domain 3<br>Incomplete outcome data<br>(attrition bias)	Low	All the specified outcome data was available
Domain 4<br>Blinding of outcome assessment<br>(detection bias)	Low	The outcome was uptake and proportion measurement was<br>the same in the groups
Domain 5<br>Selective reporting<br>(reporting bias)	Some concern	No published protocol

Gizaw et al, 2019 Ethiopia **Risk of Bias:** low

Cluster RCT

**Bias**	**Authors' judgement**	**Support for judgement**
Domain 1<br>Randomization<br>(selection bias)	Low	They described the randomisation process clearly
Identification of participants	Low	No baseline imbalances to suggest i bias<br>in the identification of participants
Domain 2<br>Blinding of participants and personnel<br>(performance bias)	Low	Participants in each cluster was meant to not know<br>about the other clusters, use of bufferzone<br>Intention to treat analysis used
Domain 3<br>Incomplete outcome data<br>(attrition bias)	Low	No missing outcome data
Domain 4<br>Blinding of outcome assessment<br>(detection bias)	Low	Outcome assessors prob didnt effect the outcome data
Domain 5<br>Selective reporting<br>(reporting bias)	Low	A published protocol exists

Lazcano et al, 2011 Mexico Risk of bias: some concern

Bias	Authors' judgement	Support for judgement
Domain 1<br>Randomization<br>(selection bias)	Low	Randomisation described
Domain 2<br>Blinding of participants and personnel<br>(performance bias)	Some concerns	The nurses were masked to the participants allocation when they went for their assigned visits.<br>Participants were offered cytology and hence moved to the control group if they weren't at home when the visit ccured. They however calculated using an intention to treat analysis.
Domain 3<br>Incomplete outcome data<br>(attrition bias)	Low	Diagram that clarifies the uptake (our outcome)
Domain 4<br>Blinding of outcome assessment<br>(detection bias)	Low	Proportions were used
Domain 5<br>Selective reporting<br>(reporting bias)	Low	Published protocol
