# Supplementary figures and images for: HPV self-sampling versus healthcare provider collection on the effect of cervical cancer screening uptake and costs in LMIC: a systematic review and meta-analysis
Source: Syst Rev. 2023 Jun 22;12:103. doi: 10.1186/s13643-023-02252-y (PMC10286394; doi:10.1186/s13643-023-02252-y)

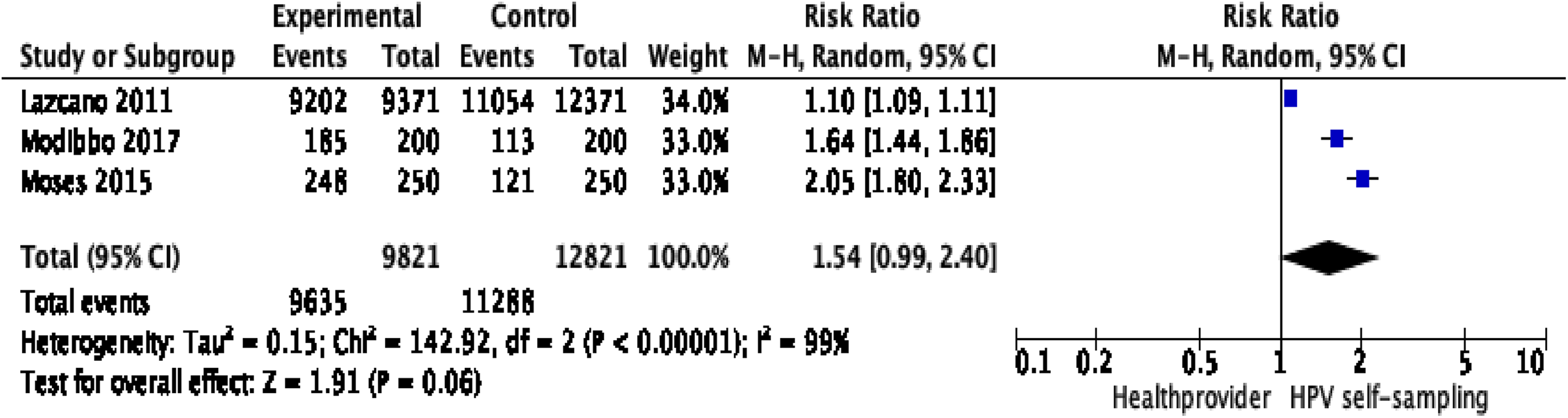

Supplement: Supplementary file 4 — Additional file 4. Sensitivity analysis — excluding cluster RCT. [file 13643_2023_2252_MOESM4_ESM.tif]
